# Nano-Ag Particles Embedded in C-Matrix: Preparation, Properties and Application in Cell Metabolism

**DOI:** 10.3390/ma15175826

**Published:** 2022-08-24

**Authors:** Sylwia Terpilowska, Stanislaw Gluszek, Elzbieta Czerwosz, Halina Wronka, Piotr Firek, Jan Szmidt, Malgorzata Suchanska, Justyna Keczkowska, Bozena Kaczmarska, Mirosław Kozlowski, Ryszard Diduszko

**Affiliations:** 1 Jan Kochanowski University, Collegium Medicum, Department of Surgical Medicine with the Laboratory of Medical Genetics, IX Wieków Kielc 19A Av., 25-317 Kielce, Poland; 2 Institute of Micro- and OptoElectronics, Warsaw Technical University, Nowowiejska 15/19, 00-665 Warszawa, Poland; 3 Kielce University of Technology, Al. Tysiąclecia Państwa Polskiego 7, 25-314 Kielce, Poland; 4 Łukasiewicz Research Network, Tele and Radio Research Institute, ul. Ratuszowa 11, 03-450 Warszawa, Poland; 5 Łukasiewicz Research Network, Institute of Microelectronics and Photonics, ul. Wólczyńskiej 133, 01-919 Warszawa, Poland

**Keywords:** genotoxicity, mutagenicity, cytotoxicity, nanocomposite nano-Ag

## Abstract

The application of nano-Ag grains as antiviral and antibacterial materials is widely known since ancient times. The problem is the toxicity of the bulk or big-size grain materials. It is known that nano-sized silver grains affect human and animal cells in some medical treatments. The aim of this study is to investigate the influence of nano-Ag grains embedded in a carbonaceous matrix on cytotoxicity, genotoxicity in fibroblasts, and mutagenicity. The nanocomposite film is composed of silver nanograins embedded in a carbonaceous matrix and it was obtained via the PVD method by deposition from two separated sources of fullerenes and silver acetate powders. This method allows for the preparation of material in the form of a film or powder, in which Ag nanograins are stabilized by a carbon network. The structure and morphology of this material were studied using SEM/EDX, XRD, and Raman spectroscopy. The toxicology studies were performed for various types of the material differing in the size of Ag nanograins. Furthermore, it was found that these properties, such as cell viability, genotoxicity, and mutagenicity, depend on Ag grain size.

## 1. Introduction

Nanomaterials based on silver with antimicrobial and antiviral properties are the subject of many studies, taking into account their preparation methods and properties. In particular, stable silver nanoparticles (AgNPs) are interesting since they can be applied in many fields of science, as well as in biotechnical and medical materials. These Ag nanoparticles can be prepared in many ways using biological, chemical, and physical methods.

Transition metals, such as Ag, can lose one electron from its orbitals. Silver (Ag) has 47 electrons, that are arranged in the configuration [Kr]4d_10_5s_1_. This electron configuration, with a single electron in the highest, occupied *s* subshell over a filled d-subshell, results in the numerous properties of this metal [1,2]. The crystalline structure of silver is *ffc* type with coordination number 12, where only the single 5s electron is delocalized. The d-subshell of Ag is fully occupied, its main +1 oxidation state exhibits relatively few properties of transition metals from groups 4 to 10, forming rather unstable organometallic compounds. Silver does not react with air, even at high temperatures. It has also been observed that silver reacts with sulfur and its compounds; however, it does not react with halogens, with the exception of fluorine gas. Moreover, silver does not react with non-oxidizing acids; however, it dissolves in hot concentrated sulfuric acid, and especially in the presence of hydrogen peroxide. Furthermore, silver dissolves in aqueous solutions of cyanide [3,4].

Silver, its salts, and vapor are highly toxic for the human organism and may cause many illnesses, but when it is stabilized in the form of nanoparticles in some network, it can be used as a weapon against many bacteria and viruses. Silver nanoparticles in the size of 10–100 nm are used medicinally in antibacterial, antivirus, and antifungal media [5,6,7,8].

With the development of nanotechnology, silver nanoparticles have become one of the most in-demand nanoparticles owing to their exponential number of uses in various applications. Silver nanoparticles (AgNPs) are used in medicine, medicinal devices, pharmacology, biotechnology, electronics, engineering, energetics, magnetic fields, and also in environmental remediation [9]. Moreover, due to their highly effective antibacterial activity both in solution and in components, AgNPs have gained popularity in the industrial sector, including textiles, food, consumer products, medicine, etc. Currently, AgNPs are extensively used in healthcare products, hygiene products, the food industry, paints, cosmetics, medical devices, sunscreen, bio-sensors, clothing, and electronics [10,11,12,13,14,15,16,17].

The role of Ag and Ag^+^ release in the mechanism was observed in numerous studies (e.g., [18,19,20]). The surface properties of Ag nanoparticles have an impact on their potency, as they influence both physical (aggregation, affinity 25 for bacterial membrane, etc.) and chemical (dissolution, passivation, etc.) phenomena. Several studies observed a high level of reactive oxygen species (ROS) in cells treated with AgNPs [21,22,23,24]. AgNPs may disrupt the membrane integrity on local bacterial membrane, increase the production of intracellular ROS, and inactivate energy-dependent metabolism. The mechanistic understandings on the physical contact and chemical interactions with bacterial intracellular ROS develop the potential of using this nanohybrid for the control of antibiotic-resistant bacteria. The use of metal nanoparticles provides an opportunity for novel antiviral therapies. Since metals may attack a broad range of targets in the virus, there is a lower possibility to develop resistance as compared to conventional antivirals. Size dependence of the AgNPs on antiviral activity was observed: Antiviral activity was generally stronger with smaller AgNPs in the composites [25,26,27,28,29,30,31,32,33,34]. On the other hand, the use of AgNPs carries a series of unpredictable concerns regarding their interaction with biological systems [35].

There were many efforts to elaborate on the method of preparation of stabilized AgNPs, which are protected against chemical reactions destroying NPs or changing their superficial properties. The chemical methods lead to preparation of metal nanoparticles in the form of a powder. The efficiency of chemical processes is high and preparation cost is low. The main problem is the aggregation of the prepared nanoparticles into big particles and the toxicity of the products formed during the chemical process. Physical methods are more expensive and complicated, but they allow for the preparation of silver NPs in many forms.

In this paper, we present a method of preparation of silver NPs placed in a carbonaceous matrix that stabilizes silver NPs and prevents their aggregation, as well as the chemical reaction taking place on the interface of NPs and their surroundings. Our method was patented in the year 2007 [36]. This patent was applied for the preparation of nanocomposite silver NPs—carbonaceous film that was described in the paper [37]. This film was obtained using PVD in the following way. The process was performed in a dynamic vacuum with the pressure of 10^−5^ mbar from two separated sources, one containing C_60_ fullerenes and the other with silver acetate. In this paper, we prepared the film on different substrates (glass, Mo foil, Si wafer). We studied some properties of the prepared films and found dependence between these studied properties using Raman spectroscopy, electron microscopy, and X-ray diffraction, and their cyto-, genotoxicity, and mutagenicity. The results of the above toxicological methods were correlated with characterization results and we discussed this dependence.

## 2. Materials and Methods

### 2.1. Material Preparation

The carbonaceous—silver nanoparticles powders were obtained on Si wafer substrate using PVD technology. The technological processes were performed in dynamic vacuum at 10^−5^ mbar. A quartz distancer with the shape of a roller with a diameter of 90 mm and a length of 100 mm was applied. The Si substrate was placed on the distancer. The evaporation was realized simultaneously from two separate sources: The first containing C_60_ fullerenes (Sigma-Aldrich, St. Louis, MO, USA, purity > 99.5%) and the second containing CH_3_COOAg silver acetate (Sigma-Aldrich, purity 99.99% with trace metals basis). The parameters of the process were: Intensity of current through silver acetate source 48A and through fullerene source 22A. The duration time of the process was 8 min. The temperature of the substrate during the process was 64 °C. The powder was obtained by removing a deposited film from the Si substrate and glass distancer. Moreover, we prepared samples in the same conditions as the nanocomposite carbonaceous—silver nanoparticle films (C-nAg) on an alumina tape substrate. These films were used for structural and molecular characterization of the material. The film thickness was ~150 nm.

### 2.2. Description of Material Characterization Methods

The obtained material in the form of a film was studied with scanning electron microscopy (SEM) with energy-dispersive X-ray spectrometry (EDX), X-ray diffraction (XRD), and Raman spectroscopy (RS). The material in the form of a powder was used for toxicological studies.

#### 2.2.1. Scanning Electron Microscopy/X-ray Energy Dispersion

The topography and morphology of the films were studied with scanning electron microscopy (SEM) with energy dispersive X-ray (EDX) composition analysis. These investigations were performed with the JEOL JSM-7600F field emission scanning electron microscope. For this purpose, the film samples were prepared on an alumina tape substrate. Observations of the surface topography were carried out at 5 keV incident electron beam energy. The microscope was equipped with an energy dispersive X-ray (EDX) spectrometer using X-MaxN 150 Silicon Drift Detector (Oxford Instruments). Due to the small thickness of the C-nPd layer, the quantitative analysis was carried out at a 5 kV accelerating voltage.

X-ray diffraction (XRD) studies were performed with a Rigaku SmartLab 3 kW diffractometer at room temperature in the θ/2θ scanning mode with CuKα radiation and Si:Li semiconductor detector. Diffraction measurements were performed in grazing incidence primary beam geometry (GIXD).

#### 2.2.2. Raman Spectroscopy

The Raman spectra measurements were carried out using an automated micro-Raman Nicolet Almega XR spectrometer (Thermo Scientific, Waltham, MA, USA). The measurements were carried out in ambient temperature for the excitation wavelength of 532 nm. The laser beam had a power of ~2.5 mW. Acquisition of spectra was performed for the spectral range from 100 to 4000 cm^−1^. Measurements were performed in micro mode using an integrated BX51 microscope (Olympus).

### 2.3. Sample Preparation for Biological Studies

Obtained samples were placed in a medium prepared in the following concentrations: 1.5 g/mL of medium for P1 and 2 g/mL for P2. The P1 specimen was obtained by removing the C-Pd film from the Si substrate, the P2 specimen was obtained by removing the C-Pd film from the distancer. We did not measure the content of Ag in the specimens due to the following reasons: They were in the form of a powder with a very low amount, and it was not possible to investigate it using EDX or X-ray fluorescence methods. Taking into account the technological process mechanism, we speculate that specimen P1 could contain less silver than specimen P2. This speculation could be confirmed by an observation that dilution in the medium of the first specimen was easier than the dilution of P2 in this medium.

#### 2.3.1. Cell Culture and Treatment

L929 cell line was obtained from the European Collection of Authenticated Cell Cultures (Sigma Chemical Co., St. Louis, MO, USA). The cells were cultured as adherent monolayers in plastic tissue culture dishes in Dulbecco’s Modified Eagle Medium (DMEM) (American Type Culture Collection, Manassas, VA, USA) supplemented with 10% (*v*/*v*) heat-inactivated Fetal Bovine Serum (American Type Culture Collection, Manassas, VA, USA) and Antibiotic Antimycotic Solution (1 mL per 100 mL of cell culture medium) (Sigma Chemical Co., St. Louis, MO, USA). The cells were maintained at 37 °C in a humidified incubator in an atmosphere containing 5% of CO_2_.

Samples P1 and P2 were dissolved in a complete cell culture medium in the following concentrations: 1.5 g/mL for P1 and 2 g/mL for P2. The final concentrations were obtained by dilution in the completed cell culture medium.

#### 2.3.2. Cytotoxicity and Genotoxicity Assays

The most common applications of cytotoxicity testing techniques are based on cell viability, measuring staining with MTT or NRU assays. Cytotoxicity can be mediated by different mechanisms. Inside the cells, many different organelles can be involved in toxicity regulation, which is why many researchers recommend using different tests for the evaluation of cytotoxicity. From this standpoint, the LDH assay is the third test that is recommended for the evaluation of cytotoxicity on the same cells, at the same time as MTT and NRU assays [38].

In the presented study, the genotoxicity in fibroblasts was measured with the use of two assays: The micronucleus test and the comet test. The comet assay is a technique for the measurement of DNA damage. Under an electrophoretic field, damaged DNA (containing single- and double-stranded breaks or alkali-labile sites) is separated from undamaged DNA, yielding a classic “comet tail” shape, which can be observed under the microscope [39].

The in vitro micronucleus test detects the formation of micronuclei in cells resulting in chromosome breakage or loss. Micronuclei are small fragments of chromosomes or whole chromosomes (surrounded by a membrane), which are unable to attach to the spindle at mitosis and form small bodies inside the cell. In this test, Cytochalasin B is used to block cell division without nuclear division blocking, resulting in cells with two or more nuclei. Then, the number of cells with micronuclei can be counted, giving a representation of the genotoxicity of the test item [40].

The Ames test principle is based on the hypothesis that different mutagens lead to mutations in many genes, including some of those mutations that reverse the ability to synthesize histidine (reverse mutations). After incubation of his- *Salmonella typhimurium* with the tested materials, in the presence and absence of exogenous metabolic activation (S9 fraction), the bacteria are transferred on a medium without histidine. After the incubation, the number of revertant colonies per plate should be scored. The ratio is calculated between the surviving control bacteria not exposed to the mutagen and the surviving experimental bacteria that were exposed to the mutagen.

There are several different strains of *S. typhimurium* with different mutations in their DNA:

- TA100 containing the same base pair substitution mutation.
- TA98 containing the same Frameshift mutation [41].

Recommended criteria for the evaluation of bacterial mutagenicity data (Ames test) comply with Mutation Research/Genetic Toxicology and Environmental Mutagenesis [42].

The studies were conducted in accordance with OECD TG 431 and 432: *OECD Guidelines for the Testing of Chemicals*, Section 4; Test No. 431*: In vitro skin corrosion, the reconstructed human epidermis (RHE) test method*; and Test No. 432: *In vitro 3T3 NRU phototoxicity test.*

To perform the MTT reduction, the LDH release, the Neutral Red Uptake, Comet, and Micronucleus assays, the cells were cultured on 96-well plates (2 × 10^5^ cells/mL) in a 100 μL complete growth medium. After 24 h, the medium was exchanged for fresh media supplemented with P1 and P2 at a dilution of 1:10, 1:100 or 1:1000. After 24 h of incubation, the MTT reduction, the LDH release, the Neutral Red Uptake (TOX-1, TOX-4, and TOX-7, respectively, Sigma-Aldrich), Comet (Oxi Select 96-Well Comet Assay Kit by Cell Biolabs, INC) assays were performed using commercially available kits, according to the manufacturer’s instructions as previously described in the literature [43]. The Micronucleus assay *in vitro* was performed according to OECD “Guideline 487: *In vitro* *Mammalian Cell Micronucleus Test*” (adopted on 26 September 2014) and PN-EN ISO 10993-3:2008. The assay was performed as it had been described in [42].

#### 2.3.3. Mutagenicity Assay

The Ames assay was performed according to OECD Guideline 471: *Bacterial Reverse Mutation Test*. Ames test was performed according to the original manufacturer’s instruction—Muta-Chromo Plate, Bacterial Strain KIT (EBPI). The assay was performed as it had been described in [42].

#### 2.3.4. Statistical Analysis of the Data

The results were expressed as mean ± SD and the data were analyzed using the *t*-Student test using Statistica software. In all cases, *p* < 0.05 was considered significant.

## 3. Results and Discussion

### 3.1. SEM and XRD

**Figure 1:**
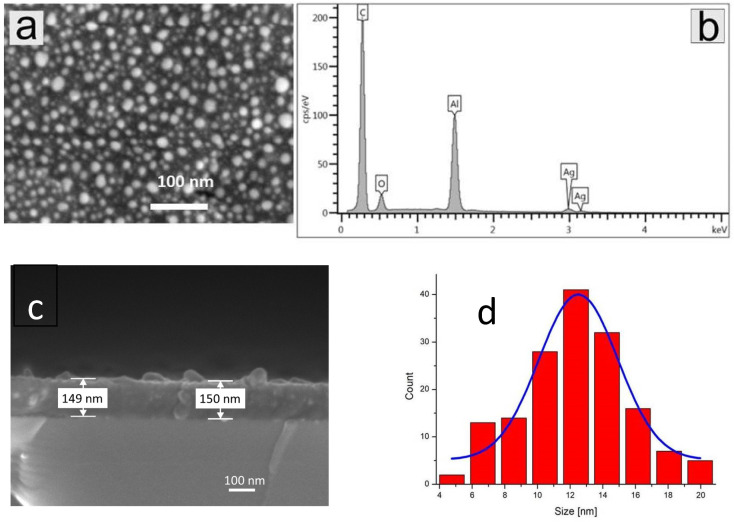
(**a**) SEM image of surface of C-nAg film on alumina substrate (equivalent to Si substrate), (**b**) EDX spectrum from this sample, (**c**) SEM image of cross-section of the film deposited on Si substrate, (**d**) statistical analysis of Ag nanograins size distribution.

SEM images of C-nAg films (sample P1) deposited on alumina and Si substrates are presented in Figure 1a–d. It should be noted that the C-nAg film covers the surface of the substrate uniformly. The highest magnification (obtained by digital processing) shows that the deposited film is composed of many very small objects with a size of about a few nm that are Ag nanocrystallites. The size distribution analysis of Ag nanograins observed in the film is presented in Figure 1d (the average size of Ag nanograin is ~12 nm, but some particle with size bigger than 20 nm is also observed).

The EDX spectrum (in region 0–5 keV) confirms the presence of Ag in the examined films (Figure 1b). The bands observed in the spectrum are attributed to C, O, Al, and Ag compounds. The band attributed to Al is connected to the substrate and the signal from Al is visible since the thickness of the films is very low (~150 nm, Figure 1d) for this exciting energy (5 kV), and the electron beam can pass through this nano Ag-C film easily to excite the substrate. The compounds C and O are connected to a carbonaceous matrix, which can be a mixture of carbonaceous and carbon oxide objects. The signal connected to the Ag compound originates from Ag nanograins. We are not able to determine the amount of particular compounds due to the very low thickness of the film and the strong influence of the Al substrate on the spectrum.

The cross-section through the film deposited on the Si wafer seen in Figure 1c shows that Ag nanograins found as bright objects are placed also within the film.

SEM studies for powder samples could not be performed since a powdered sample cannot be introduced into the vacuum chamber of a microscope column. To achieve some complementary information on the size of the silver particles in the powder sample, we performed XRD studies for samples P1 and P2.

**Figure 2:**
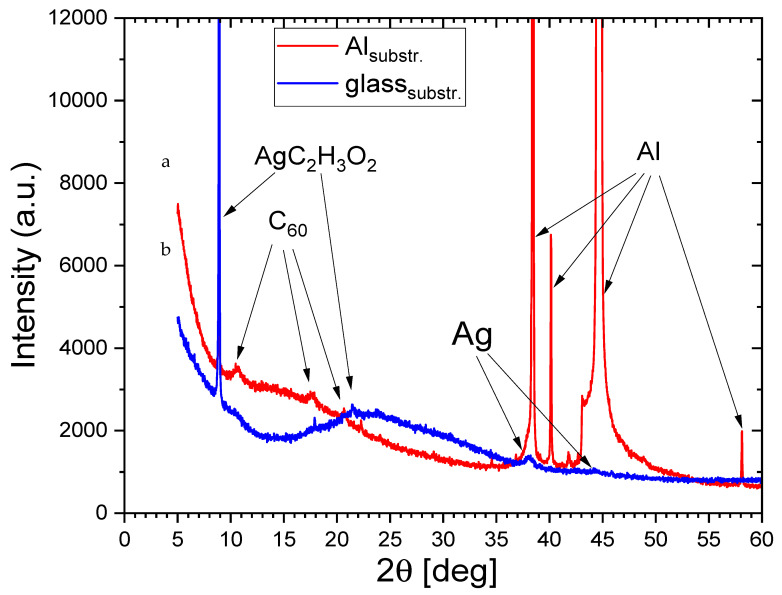
X-ray diffraction patterns for: (**a**) The P1 sample (red line); (**b**) the P2 sample (blue line).

The X-ray diffraction studies have shown a difference in the structure of the nano-Ag-C P1 and P2 samples. In the XRD pattern for the P1 sample (Figure 2 red line), in addition to the silver attributed peaks, we also observe peaks coming from the silver acetate and C_60_ fullerite. The identified peaks are marked with arrows. The XRD spectrum of the P2 sample (blue line) shows the peaks coming from the same compounds as for the P1 sample. The Ag peak’s intensity for the P2 sample indicates the small size of these grains (considerably lower than those observed in P1 sample). The XRD results (Ag peak intensity and width) suggest that Ag nanograins in sample P1 are bigger than in sample P2. We are not able to calculate the size of Ag nanograins due to the covering of Ag originating peaks and Al peaks, which are very strong and preclude determination of the intensity and half width of Ag connected peaks. The structure of Ag nanograins in sample P1 is of the *fcc* type of crystal structure with a little shift of lattice parameters toward a bigger value than the known lattice parameter for bulk silver material (0.4 nm). This effect is connected with the very small size of the grains.

The comparison of SEM and XRD results allows us to state that the P1 sample (film) has Ag grains sized between 10 and few tens of a nanometer, which is reflected in strong Ag peak formation in the XRD diffraction pattern, while for sample P2 this peak is considerably weaker and broader, which is connected to the significantly lower size of Ag grains in P2 sample.

### 3.2. Raman Spectroscopy Results

Raman spectra allow us to determine the structure of the carbonaceous matrix. These spectra look very similar for both types of samples (P1 and P2).

It is known from the literature that in a typical Raman spectrum of graphite and graphite-like material there are three characteristic bands: D, G, and 2D. The G-band (1500–1600 cm^−l^) is connected to the presence of sp^2^ carbon hybridization of bonded carbon atoms densely packed in the planar hexagons sheet, and the D-band (~1350 cm^−l^) is due to the disorder induced in the ring, i.e., a structure that does not support π-electrons in forming a ring (conjugated cyclic) system implies the absence of the D-band [43]. These D and G bands were observed and described in detail for graphite and graphite-like materials by Tuinstra and Koenig [44].

Raman spectra of different carbonaceous materials possessing D and G bands allow for the calculation of the I_D_/I_G_ ratio. The deconvolution of the spectrum in the region of bands D and G leads to the determination of band widths and intensities. The deconvolution results depend on the fitting model, in which the bands’ shape is chosen. In [45], the role of the fitting model (e.g., choice of Gauss, Voight, asymmetric band shape) was discussed. The authors suggest that due to the large variety of Raman spectrum shapes, the Lorentzian shape is significantly restrictive, and the Voigt shape (a mixing of Lorentzian and Gaussian shapes) is considerably more suitable. In this paper, we present the results of deconvolution using the Voigt band shape. In [44], the formula binding I_D_/I_G_ with exciting laser energy E_L_ and the f(L_a_), a function of the in-plane crystallite size La (size of the area with carbon atoms densely packed in the planar hexagons sheet), is shown as follows:

$$I_{D}∕I_{G}=\frac{E_{L}}{f\left(\mathrm{La}\right)}$$

where I_D_ and I_G_ denote intensities of bands D and G, respectively.

Depending on the applied model, f(La) can be described in a different way, but generally, the information about the ordered area can be obtained. It is known that for low ordered carbon material I_D_/I_G_ 1 and higher, when the La size of the ordered area increases, then the ratio I_D_/I_G_ decreases and is significantly lower than one [46].

**Figure 3:**
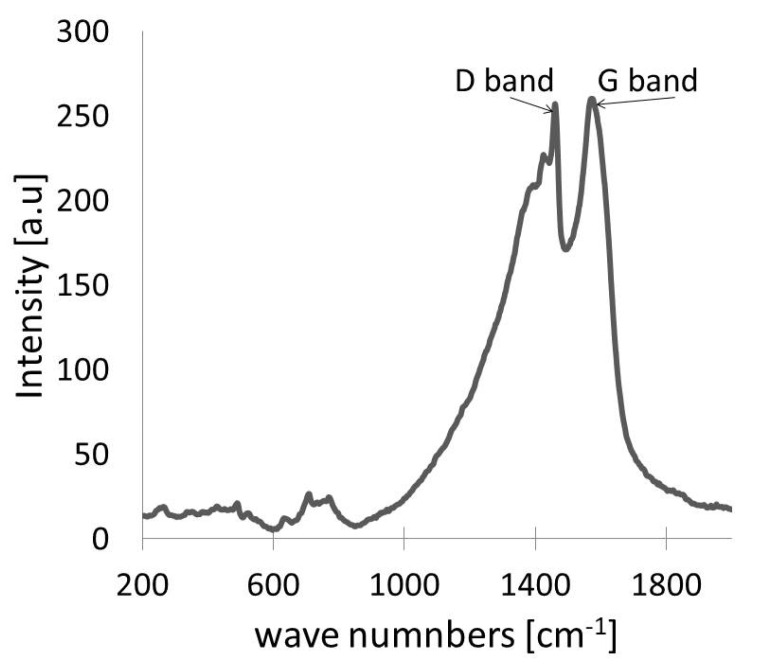
Raman spectrum of the prepared nano-Ag-C film deposited on alumina substrate.

The Raman spectrum of a typical nano-Ag-C film sample is presented in Figure 3. Two highest bands placed at 1308 and 1580 cm^−1^ were found. These bands can be respectively attributed to the D and G bands observed for graphite structure. In accordance with [45,46,47], information about the in-plane graphite-like crystallite size L_a_ can be obtained. The analysis of the shape and position of these two bands (D and G) can lead to complementary information about the ordered carbon areas. A formula binding the intensity of bands D and G (I_D_/I_G_) with exciting laser beam energy E_L_ and the f(L_a_) function of the in-plane crystallite size L_a_ was determined.

**Figure 4:**
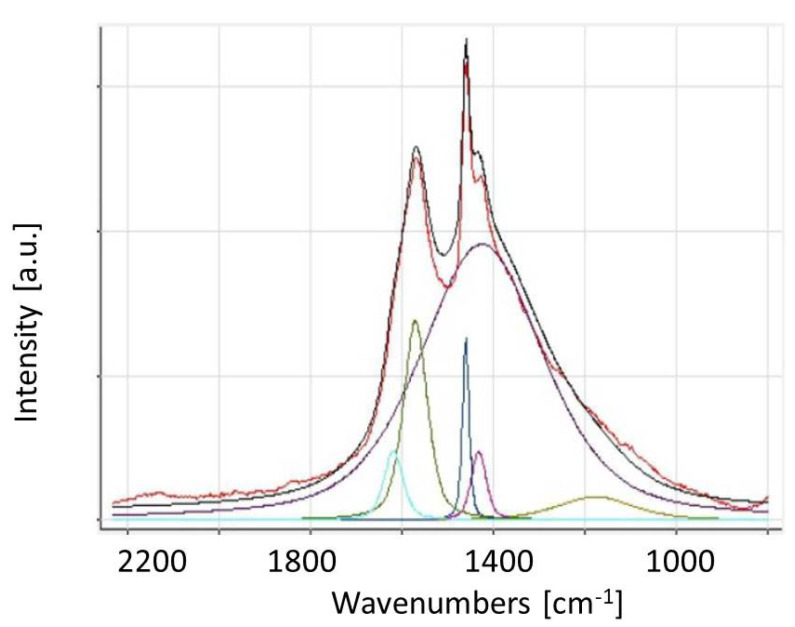
The result of the decomposition of the Raman spectrum of nano-Ag-C film in the area of 800–2200 cm^−1^. The line colors are as follows: Black line—experimental spectrum; brown line—calculated spectrum; other colors—arbitrary assigned by Peak Spectroscopy Software.

The result of the fragment of Raman spectrum decomposition (for the spectral area 900–2000 cm^−1^) is shown in Figure 4.

The shape analysis of the spectrum of the nano-Ag-C film was performed using Peak Spectroscopy Software and the Voigt profile was used for the line shape. The results of this analysis showed that for the spectrum in the region 800–2200 cm^−1^, there are six bands placed at 1178, 1426, 1433, 1461, 1571, and 1619 cm^−1^. Two of these bands are the D-band (1426 cm^−1^) and the G-band (1571 cm^−1^). The bands at 1433 and 1461 cm^−1^ could be connected to distorted C_60_ molecules’ vibrations. This band at 1461 cm^−1^ could be connected to the shifted mode assigned to the tangential stretching mode of 5-fold pentagon carbons in isolated C_60_ molecule, as well as in fullerene powder or film. The band at 1433 cm^−1^ is usually observed in C_60_ molecules interacting with other C_60_ molecules (e.g., in solids) or other molecules or surfaces [48]. The very weak bands placed at 1178 and 1619 cm^−1^ can be connected to palladium acetate molecule vibration [49]. This residual palladium acetate originates from a technological process.

To calculate the I_D_/I_G_ ratio, a deconvolution of a spectrum in the spectral region 900–2000 cm^−1^ was performed, which is equal to 1.38 (see Table 1). Of note, the ratio I_D_/I_G_ is a measure of disordered carbon and normally expressed (sp^2^/sp^3^) carbon ratio [47]. The increase in the value indicates the relative increase in sp^2^ domains and decrease in average crystallite size [44]. It is easy to find that the ratio is inversely proportional to the crystallite size L_a_ [50]:

L_a_ = (2.4 × 10^−10^) λ^4^ _laser_ (I_D_/I_G_)^−1^

In our experiment, the laser wavelength was 532 nm, thus the calculated crystallite size L_a_ was 13.92 nm. In [50], using the same formula, the crystallite domain size of graphene calculated for graphene Ag nanoparticle hybrids has been found to be 18 nm.

The nature of the Raman band in our sample is a little different compared to the monolithic graphene. The position of the G-band is shifted a little during interaction with Ag particles. This can indicate that the vibrational nature of the small graphitic domain has not been significantly affected by the presence of the Ag nanoparticles. On the other hand, the position of the D-band has undergone a blue shift compared to pure graphene. This major shift indicates that the vibrational energy due to stretching of sp^2^ -bonded carbon is increased in our sample. It is possible that this shift is connected with the position of the Ag nanoparticles on the surface of graphene. The authors of [50] suggested that Ag occupies the top of the carbon atom in graphene. Moreover, they reported that the major shift of the D-band may be due to the larger distortion generated by this Ag occupation. The enhancement in the ratio (I_D_/I_G_) can indicate disorder and that, for example, the new graphitic domains are bigger in number, but smaller in size.

### 3.3. Cyto-, Genotoxicity, and Mutagenicity

**Figure 5:**
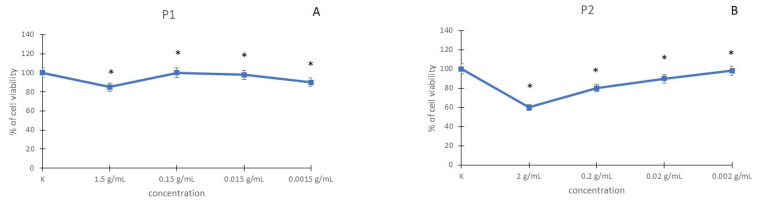

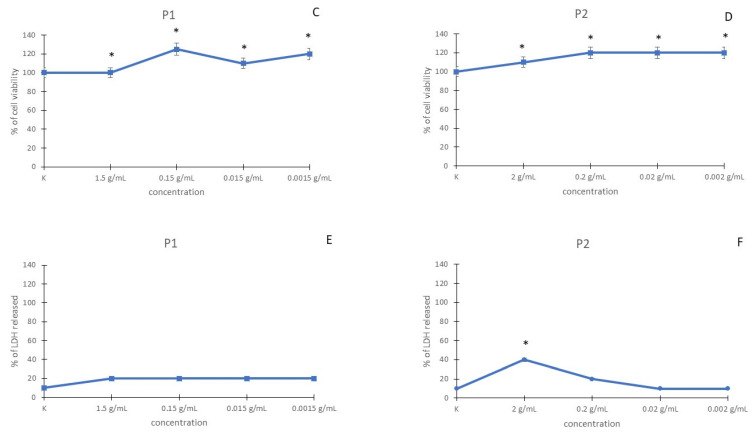
Cytotoxic effect of P1 and P2 in L929 cell line, detected with the MTT reduction assay (**A**,**B**), with the NRU assay (**C**,**D**), and with the LDH release assay (**E**,**F**). * *p* < 0.05, significance of difference compared with control.

L929 cells were exposed to P1 at final concentrations of 1.5, 0.15, 0.015, and 0.0015 g/mL and P2 at final concentrations of 2, 0.2, 0.02, and 0.002 g/mL. The cytotoxicity was determined with the MTT reduction, the LDH release, and the Neutral Red Uptake assays. The results are presented in Figure 5.

In the MTT assay, after incubation of fibroblasts with P1 at a concentration of 1.5 g/mL, the decrease in cell viability was observed (10%) when compared with control cells. Other used concentrations of P1 were nontoxic. Cytotoxicity was observed in cells incubated with P2 at a concentration of 2 g/mL. In the other used concentrations of the P2 (0.2, 0.02, and 0.002 g/mL), the increase in cell viability was observed (Figure 5A,B). The IC50 dose was not determined in the MTT reduction test.

The results using the NRU assay in P1 and P2 in L929 cells are shown in Figure 5C,D. Both used P1 and P2 in all used concentrations were nontoxic on fibroblasts. The IC50 dose was not determined in the NRU test.

Cell viability was also determined by the LDH release assay. Figure 5E,F presents the dose-response curve for the LDH assay after incubation with P1 and P2. In fibroblasts incubated with P2 at a concentration of 2 g/mL, the increase in LDH leakage was observed. In the other used concentrations of the P2 (0.2, 0.02, and 0.002 g/mL), the increase in LDH leakage was not observed. In the L929 cell line, P1 did not cause any increase in LDH release. The IC50 dose was not determined in the LDH release test.

**Figure 6:**
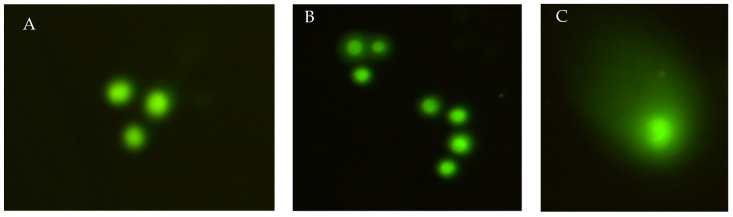
Typical fluorescence microscopic images of L929 cells exposed to P1 (**A**) and P2 (**B**) and comets formed after exposure of P1 (**C**).

Genotoxicity was assessed both by analyzing the induction of micronuclei formation by micronucleus formation assay and DNA damage by comet assay in L929 cells. The results using the comet assay in P1 and P2 in L929 cells are shown in Table 2. The results obtained from fibroblasts show that both used specimens are nontoxic. The typical cells in the used specimens are presented in Figure 6A,B. Single comets induced after exposure to P1 are shown in Figure 6C.

**Figure 7:**
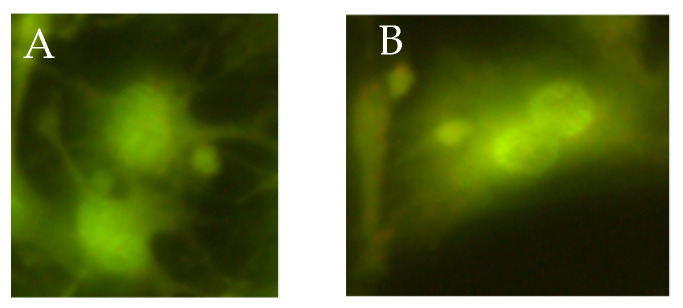
Fluorescence microscopic images (micronuclei) of the acridine orange stained L929 cells exposed to P1 (**A**) and P2 (**B**).

The micronucleus assay performed with the use of all concentrations of both specimens does not show statistically significant induction of chromosomal aberrations (Table 3). Single micronuclei induced after exposure to P1 and P2 are shown in Figure 7.

Mutagenicity was assessed using the Ames assay. A tested substance is considered not mutagenic in the test if the total number of revertants in tester strain TA100 is not greater than twice the concurrent control, and the total number of revertants in tester strain TA98 is not greater than thrice the concurrent vehicle control. In both tested specimens (P1 and P2), the increase in the number of reverse mutations with and without metabolic activation was not observed (Table 4).

## 4. Conclusions

Nano-Ag-C material is a very attractive nanomaterial for commercial applications. This material can be widely used for antimicrobial and biomedical products. In this paper, we showed:

1. The new and original material that we elaborated, which can be obtained in the form of a film as well as in the form of a powder. The material obtained in this study stabilizes the silver nanograins preventing their aggregation, which was shown by SEM studies.
2. For film samples, the average size of Ag nanograins was 12 nm, but nanograins bigger than 20 nm in diameter were also observed. The size of Ag nanograins is significantly lower in a powder sample.
3. Raman spectroscopy results showed that the carbonaceous matrix is composed of graphene-like domains with a size of ~14 nm. The Raman bands confirm the existence of C_60_ molecules interacting with other C_60_ molecules and/or with metal (Ag) nanograins.
4. The influence of the size of silver nanograins (i.e., of the synthesis method) on cytotoxicity, genotoxicity, and mutagenicity was found. Mitochondria may be the first part of the cell to be affected by both P1 and P2 specimens. The damage of the cell membrane and lysosomes follows mitochondria disruption.
5. Both specimens (P1 and P2) used in this study do not induce genotoxicity and mutagenicity. Based on all toxicological assays used in this study, it can be concluded that P1 and P2 were nontoxic in the L929 cell line.

Our results allow for planning the practical use of our material in bio- and medical applications.

## Figures and Tables

**Table 1:** The results of the Raman spectrum deconvolution for nano-Ag-C film for the D and G bands.

Material	D-Band		G-Band		I_D_/I_G_
	Wavelength ω [cm^−1^]	Full Band Half Maxim um Γ_1/2_ [cm^−1^]	Wavelength ω [cm^−1^]	Full Band Half Maximum Γ_1/2_ [cm^−1^]	
n-Ag-C film	1426	160	1571	31	1.38

**Table 2:** Percentage of DNA tail after incubation with P1 and P2 in L929 cell line detected with comet assay.

	Concentration	Percentage Tail DNA [%]
K		0
P1	1.5 mg/mL	4
	0.15 mg/mL	3
	0.015 mg/mL	1
	0.0015 mg/mL	1
P2	2 mg/mL	5
	0.2 mg/mL	4
	0.02 mg/mL	2
	0.002 mg/mL	1

**Table 3:** Frequency of micronucleated binucleated cells after incubation with P1 and P2 in L929 cell line detected with micronucleus assay.

	Concentration	BNMN [‰]
K		0
P1	1.5 mg/mL	10
	0.15 mg/mL	3
	0.015 mg/mL	1
	0.0015 mg/mL	1
P2	2 mg/mL	11
	0.2 mg/mL	4
	0.02 mg/mL	1
	0.002 mg/mL	0

**Table 4:** Bacterial reverse mutation results (mean revertant numbers per plate) in *Salmonella typhimurium* TA98 and TA100 strains after incubation with P1 and P2 detected with the Ames assay.

	Concentration	TA98		TA100	
		−S9	+S9	−S9	+S9
K		5 ± 1	4	4	4
NaN_3_		-	-	98	97
2-NF		98	97	-	-
P1	1.5 mg/mL	10 ± 1	9 ± 1	2	3
	0.15 mg/mL	5 ± 1	7 ± 1	2	3
	0.015 mg/mL	5 ± 1	7 ± 1	2	3
	0.0015 mg/mL	5 ± 1	6 ± 1	2	3
P2	2 mg/mL	16 ± 1	14 ± 1	16	15
	0.2 mg/mL	16 ± 1	13 ± 1	14 ± 2	13 ± 2
	0.02 mg/mL	20 ± 2	12 ± 1	14 ± 2	12 ± 2
	0.002 mg/mL	20 ± 2	10 ± 1	14 ± 2	10 ± 1

## Data Availability

Not applicable.
